# *Mms19* is a mitotic gene that permits Cdk7 to be fully active as a Cdk-activating kinase

**DOI:** 10.1242/dev.156802

**Published:** 2018-01-15

**Authors:** Rishita Narendra Nag, Selina Niggli, Sofia Sousa-Guimarães, Paula Vazquez-Pianzola, Beat Suter

**Affiliations:** Institute of Cell Biology, Department of Biology, University of Bern, 3012 Bern, Switzerland

**Keywords:** Mitotic gene, Mms19, Xpd, Cdk-activating kinase, *Drosophila* development

## Abstract

*Mms19* encodes a cytosolic iron-sulphur assembly component. We found that *Drosophila Mms19* is also essential for mitotic divisions and for the proliferation of diploid cells. Reduced *Mms19* activity causes severe mitotic defects in spindle dynamics and chromosome segregation, and loss of zygotic *Mms19* prevents the formation of imaginal discs. The lack of mitotic tissue in *Mms19^P/P^* larvae can be rescued by overexpression of the Cdk-activating kinase (CAK) complex, an activator of mitotic Cdk1, suggesting that Mms19 functions in mitosis to allow CAK (Cdk7/Cyclin H/Mat1) to become fully active as a Cdk1-activating kinase. When bound to Xpd and TFIIH, the CAK subunit Cdk7 phosphorylates transcriptional targets and not cell cycle Cdks. In contrast, free CAK phosphorylates and activates Cdk1. Physical and genetic interaction studies between Mms19 and Xpd suggest that their interaction prevents Xpd from binding to the CAK complex. Xpd bound to Mms19 therefore frees CAK complexes, allowing them to phosphorylate Cdk1 and facilitating progression to metaphase. The structural basis for the competitive interaction with Xpd seems to be the binding of Mms19, core TFIIH and CAK to neighbouring or overlapping regions of Xpd.

## Abstract (precis)

**Summary:** Interaction studies demonstrate that Mms19 forms complexes with Xpd, thereby preventing Xpd-mediated repression of the mitotic kinase activity of the CAK complex and facilitating progression through mitosis.

## INTRODUCTION

Considering the countless insults that DNA and chromosomes have to sustain, it is amazing how faithfully the genome is duplicated and passed on to daughter cells during mitosis. *mms19* was first identified in *Saccharomyces cerevisiae* as a gene that is necessary for repairing alkylated DNA and for the removal of ultraviolet light-induced pyrimidine dimers by the nucleotide excision repair (NER) pathway (Prakash and Prakash, 1977, 1979). Extracts from yeast cells mutant for *mms19* showed impaired RNA polymerase II transcription, a defect that could be corrected by the addition of the TFIIH transcription complexes to the extract, but not by purified Mms19 (Lauder et al., 1996). Reduction of *MMS19* activity in higher eukaryotes causes additional phenotypes such as defective mitotic spindles and chromosome segregation defects, extended telomeres and defects in methionine synthesis (Askree et al., 2004; Ito et al., 2010; Lauder et al., 1996). From this, it appears that *MMS19* might have diverse functions in eukaryotes.

MMS19 physically interacts with proteins of the cytoplasmic iron-sulphur cluster (Fe-S) assembly complex, such as Ciao1, IOP1 (NARFL), MIP18 (FAM96B) (Gari et al., 2012; Stehling et al., 2012; Ito et al., 2010). Additionally, immunopurification of human cytoplasmic complexes containing MMS19 led to the identification of 12 known Fe-S proteins, including XPD (ERCC2), RTEL1 (regulator of telomere length protein), FANCJ (Fanconi anemia protein J; also known as BRIP1), DNA Polymerase δ, and Pri2, as its interacting proteins (Gari et al., 2012; Stehling et al., 2012). The functional significance of these interactions were addressed in yeast, where the absence of *Mms19* caused a significant reduction of ^55^Fe incorporation into human XPD, which was overexpressed as a Fe-S cluster target protein. Similarly, ^55^Fe incorporation into other Fe-S cluster proteins, including Leu1, Ntg2 and Rli1, was also found to require *Mms19* (Gari et al., 2012; Stehling et al., 2012). When these target proteins did not obtain the Fe-S clusters, they displayed reduced activity and decreased protein stability. In contrast, at least in HeLa cells, knockdown of *MMS19* did not affect the activity or levels of two other known Fe-S proteins, IRP1 (iron regulatory protein 1; also known as ACO1) and GPAT (glutamine phosphoribosylpyrophosphate amido transferase; also known as PPAT) (Stehling et al., 2012). From these results it was concluded that MMS19 is involved in the assembly of Fe-S clusters of only a subset of proteins that contain such clusters. For this subset of Fe-S cluster-containing proteins, proper expression of *MMS19* is necessary for stability and protein activity. Under normal *MMS19* expression, the association of the target protein with the cytoplasmic iron-sulphur assembly (CIA) targeting complex is transient and the protein remains an apoform until the Fe-S cluster is incorporated. But in the absence of MMS19 this interaction becomes more stable as the target protein awaits the incorporation of Fe-S (Gari et al., 2012). Combined, the studies described above in yeast and human cells highlight the role MMS19 plays as part of the CIA machinery in the maturation of a subset of Fe-S-containing proteins. It contributes to their activation and indirectly affects the downstream enzymatic functions of these target proteins.

Ito et al. observed that reducing *MMS19* levels in HCT116 cells and HeLa cells strongly increased the formation of abnormal mitotic spindles (Ito et al., 2010). Aside from multipolar and monopolar spindles, chromosome segregation abnormalities increased strongly, too. This study pointed for the first time to a mitosis-specific role for MMS19 and possibly the entire MMXD complex (MMS19/MIP18/XPD). What the physiological role of this function could be and whether the mitotic defects reflect the role of MMS19 in the CIA machinery or a more direct MMS19 function in mitosis remained unknown. We set out to address this question in the *Drosophila melanogaster* model system, in which it is possible to test for mitosis-specific functions that are not dependent on the transcriptional function of the test gene. Here, we demonstrate that the mitotic function of Mms19 is essential for normal diploid cell cycles, organ growth and development. We further show that Mms19 not only has an activating role towards Xpd, but that it also functions to prevent Xpd from repressing the Cdk-activating kinase function of Cdk7 during mitosis. Mms19 thus allows Cdk7 to produce the high levels of active mitotic Cdk1 kinase that are required for cells to proceed through the first phase of mitosis.

## RESULTS

### *Mms19* is an essential gene highly expressed in ovaries, embryos and diploid larval tissue

The *Mms19^P^* allele causes lethality when homozygous or hemizygous over an unrelated deficiency (Df) chromosome. This P-element, inserted into the third exon of *Mms19*, disrupts the production of normal *Mms19* mRNA (Fig. S1). To study *Mms19* expression and Mms19 protein distribution at normal expression levels, we generated a transgenic fly line expressing Mms19 C-terminally fused to eGFP from its predicted endogenous genomic control sequences. One copy of the *Mms19::eGFP* transgene was sufficient to fully rescue the lethality of the *Mms19^P^* chromosome over an unrelated Df chromosome that removes *Mms19* (Table 1)*.* This shows that the Mms19::eGFP fusion protein is functional. The fact that two copies of *Mms19::eGFP* rescue less efficiently might point to an unknown recessive second site hit on the chromosome that carries the transgene. We also performed these rescue experiments with homozygous *Mms19^P/P^* animals (Fig. S2A). Again, we observed an efficient rescue of the lethality, even though the rescue was not 100%. This reduction might, however, point to the presence of a recessive second site hit on the *Mms19^P^* chromosome.

**Table 1.**
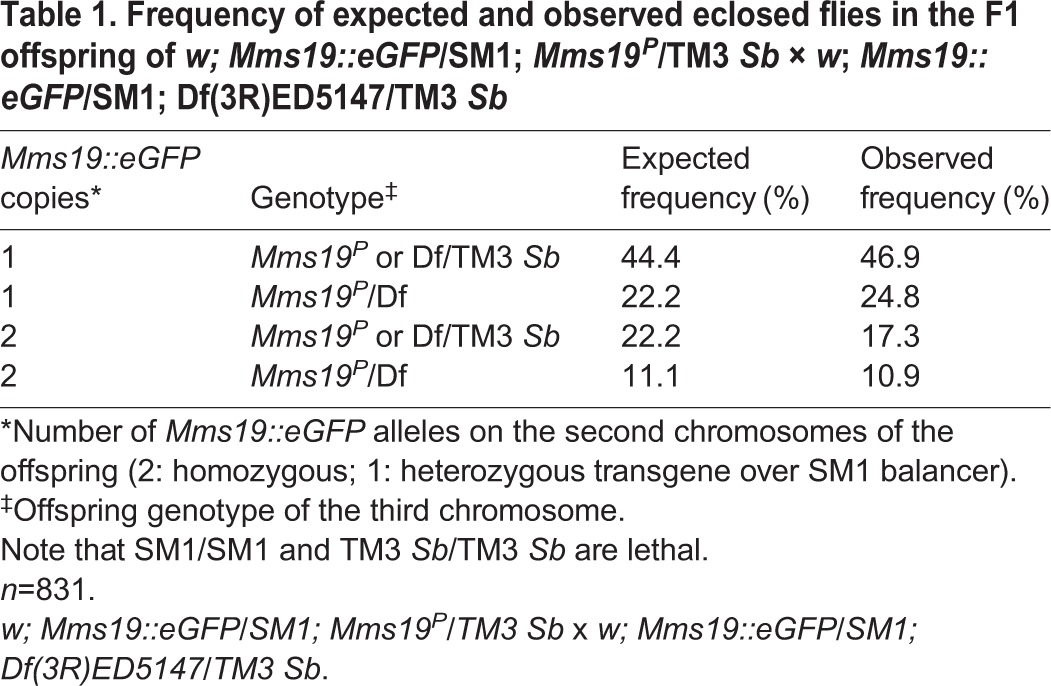
**Frequency of expected and observed eclosed flies in the F1 offspring of *w; Mms19::eGFP*/SM1; *Mms19*^*P*^/TM3 *Sb* × *w*; *Mms19::eGFP*/SM1; Df(3R)ED5147/TM3 *Sb***

FlyBase data (flybase.org) from independent projects show elevated expression of *Mms19* mRNA in young embryos, ovaries and testes, imaginal discs, and moderate expression in some other tissues and parts such as the larval CNS, salivary glands, guts and carcasses. We used the *Mms19::eGFP* line in the *Mms19^P/P^* background to confirm the expression of Mms19 in young embryos, ovaries and different tissues of third instar larvae, including brains and imaginal discs (Fig. S2B).

### *Mms19^P^* phenotype points to essential mitotic functions in diploid cells

**Fig. 1.**
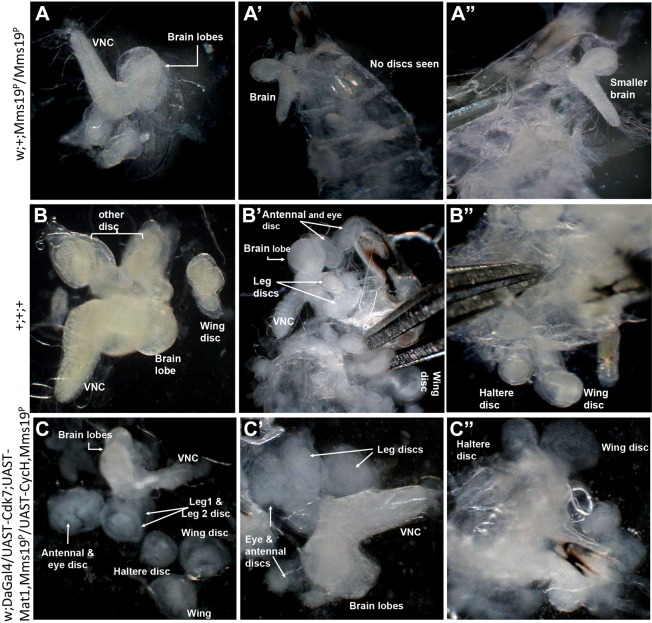
**Third instar larval phenotype.** (A-A″) *Mms19^P/P^* third instar larvae had no imaginal discs and a smaller brain. (B-B″) Imaginal discs and brains of wild-type third instar larvae. (C-C″) Dissected third instar larvae homozygous mutant for *Mms19^P/P^* and rescued by overexpression of CAK. Most discs were rescued to normal size, but their structural appearance was not wild type. All images are displayed at the same magnification. VNC, ventral nerve cord.

*Mms19^P/P^* mutants survive embryonic development possibly because of their maternal supply of functional Mms19. *Mms19^P/P^* larvae develop slowly, but reach the third instar stage. However, in contrast to wild-type larvae, mutants do not contain recognizable imaginal discs (compare Fig. 1A-A″ with B-B″) and they do not pupate. Imaginal discs are patches of cells put aside during larval stages to give rise to adult structures during pupation. These cells are diploid and undergo numerous mitotic division cycles during larval development. In contrast, large parts of the larval tissue consist of polyploid cells that have become polyploid through repeated DNA synthesis (S phase) in the absence of intervening M phases and cell divisions. These large cells with their highly polyploid nuclei are then resorbed during the pupal stage. Interestingly, these polyploid cells appear normal at the phenotypic level, indicating that they are less dependent on *Mms19.* This phenotype of the *Mms19* mutant is typical for genes involved in mitotic proliferation (Gatti and Baker, 1989).

### Loss of maternal *Mms19* causes cell cycle defects in young embryos

To study the loss-of-function phenotype of *Mms19* during mitosis, we induced homozygous *Mms19^P/P^* germline clones in females using the FRT system combined with *ovo^D^*, which eliminates wild-type germline cells (Chou and Perrimon, 1996). With this tool, all eggs laid will be derived from a germline that was homozygous mutant for *Mms19^P^*, and young embryos derived from these eggs do not obtain wild-type Mms19 protein during the early stages of development until the zygotic genes are activated. Upon fertilization by *Mms19^+/+^* fathers, 24.5% of the embryos hatched as larvae whereas upon fertilization with heterozygous *Mms19^P/+^* males only 10.5% hatched as larvae (Fig. S3). Furthermore, the maternal effect lethality could be rescued to 66.5% with two maternal copies of *Mms19::eGFP.* This indicates that maternal *Mms19* is essential for embryonic development, but about a quarter of the embryos can be rescued by one copy of zygotic *Mms19* (1 in 4 if they all receive a wild-type copy from their fathers and 1 in 8 if half of the embryos receive a wild-type copy).

**Fig. 2.**
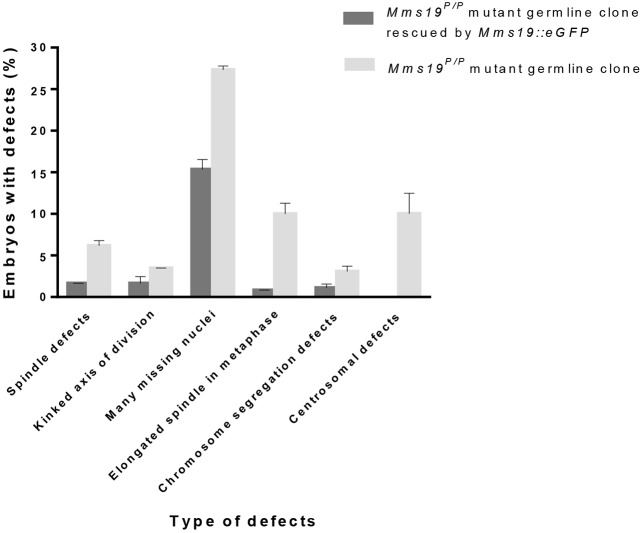
**Effects of maternal *Mms19* loss of function on young embryos.** Classification of different defects observed in young embryos derived from *Mms19^P/P^* mutant germline clones. Embryos analysed between cycles 10 and 13 exhibited severe cell cycle defects in 60% (*n*=260) of the cases, but this frequency dropped to 20% (*n*=365) when the mothers were also homozygous for the *Mms19::eGFP* fusion gene. The data shown is from three independent experiments. Error bars represent s.d.

Young *Drosophila* embryos develop in a syncytium of rapidly dividing nuclei. Confocal microscopy analysis of the syncytial division cycles of embryos derived from *Mms19^P^* eggs and heterozygous fathers revealed important mitotic functions for *Mms19* (Fig. 2). About half of the embryos reached division cycles 10-13, when cell cycle features are best visible. About 60% of these embryos showed various cell cycle defects (*n*=260), which we classified into six phenotypic classes: spindle defects, kinked axis of division, many missing nuclei, elongated spindle in metaphase, chromosome segregation defects, and centrosomal defects (Fig. 2). The expression of *Mms19::eGFP* in the *Mms19^P/P^* germline mutants rescued the defects of all six phenotypic classes at least partially, and only 20% of these embryos showed one or more of the defects in division cycle 10-13. Clearly, maternal *Mms19* is important for proper progression through mitosis in young embryos*.*

### Knockdown of Mms19::eGFP protein causes cell cycle defects and chromosomal abnormalities in young embryos

The defects observed in young embryos derived from *Mms19^P/P^* germline clones could either reflect a direct function of *Mms19* in the embryo or it could be a more indirect consequence of the absence of *Mms19* during oogenesis. To test whether *Mms19* is indeed required during embryogenesis, we decided to knock down the Mms19 protein specifically in young embryos. For this purpose, we first rescued the lethality and sterility of the *Mms19^P/P^* mutants with the already-described *Mms19::eGFP* construct under its endogenous promoter. We then knocked down the Mms19::eGFP protein (in the *Mms19^P/P^* background) using the anti-GFP nanobody-based deGradFP technique (Caussinus et al., 2012). To knock down the GFP fusion protein only in young embryos and not during oogenesis, the deGradFP construct was expressed under the control of the maternal *hunchback* (*hb*) promoter coupled with the *bcd* 3′UTR element that represses its translation during oogenesis until egg activation (P.V.-P., unpublished). Relative to the tubulin standard, only low levels of deGradFP are expressed in ovaries compared with embryos and, importantly, the Mms19::eGFP signal is not reduced in ovaries when comparing flies that have the *deGradFP* gene with those that do not carry it (Fig. S4). This is in contrast to the embryonic extracts, in which the presence of the deGradFP caused a clear reduction of the Mms19::eGFP signal relative to the loading control.

**Fig. 3.**
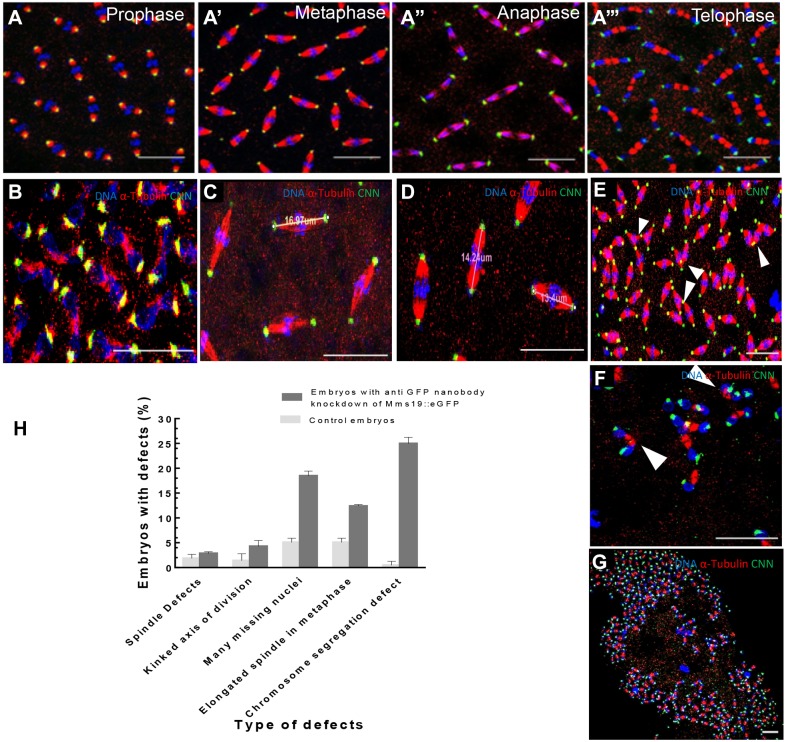
**Embryonic knockdown of Mms19::eGFP with the anti-GPF nanobody-based deGradFP technique.** (A-A‴) Overlay of control embryos expressing one copy of *Mms19::eGFP* and one copy of the deGradFP construct in the *Mms19*^+/+^ background. Embryos are in prophase (A), metaphase (A′), anaphase (A″) and telophase (A‴) of cycle 12. (B-G) Embryos with reduced Mms19::eGFP (B,C,E-G) and control embryos for comparison (D). (B) Chromosomal bridges formed as a result of chromosome segregation failure during telophase of a cycle 12. (C) Elongated metaphase spindles in a cycle 10 embryo. Elongated spindles range from 15 to 19 µm in length. The presented one is 16.97 µm long. (D) Metaphase spindles in cycle 10 of control embryos range from 12 to 14 µm. (E) Spindle defects such as formation of multipolar spindles and spindle crossovers are indicated by arrowheads. (F) Kinked axis of division of nuclei in mutant embryo during telophase (indicated by arrowheads). (G) Large numbers of missing nuclei in a mutant cycle 12 embryo. (H) Comparison of different cell cycle defects between control (*n*=218) and Mms19::eGFP knockdown embryos (*n*=493). All defects became visible during the mitotic phase of the cell cycle, when 57% of Mms19::eGFP knockdown embryos have one or more of the defects. Scale bars: 20 μm. Error bars represent s.d.

Expressed maternally in young embryos, the deGradFP system does indeed reduce Mms19::eGFP levels to less than 50% (Fig. S4; for quantification see also Fig. 5G). To study the effect of the knockdown in young embryos at the cellular level, we also used control embryos that expressed the *Mms19::eGFP* fusion construct and the deGradFP construct, but in addition contained an endogenous wild-type allele of *Mms19* (Fig. 3A-A‴). This controls for dominant effects that might be caused by degrading Mms19::eGFP by the deGradFP technique. Accordingly, we expect such control embryos to display background levels of defects that are as high as or higher than those of a true wild-type strain. Indeed, the deGradFP itself produced a slight increase in mitotic defects (Fig. 3H). As opposed to these control knockdowns, experimental knockdown of the fusion protein led to various and abundant cell cycle defects, which we classified into five phenotypic groups. Twenty-five percent of the embryos showed improper chromosome segregation and chromosomal bridges (Fig. 3H), which was evident in anaphase and telophase of the cell cycle (Fig. 3B). The mitotic spindles of mutant embryos fixed in metaphase of cycle 10 were consistently longer than control spindles (compare Fig. 3C with 3D). Apart from this, we observed higher frequencies of defects in spindle formation and dynamics (Fig. 3E), including spindle crossovers, multipolar spindles and kinked axis of division (Fig. 3F). In these cases, centrosomes often appeared misplaced from the normal axis of division (mitotic figures). Finally, the most obvious phenotype we observed was that many embryos lacked nuclei in one or more large areas (Fig. 3G). A detailed analysis of these defects revealed that 57% of Mms19::eGFP knockdown embryos (*n*=493) displayed some form of cell cycle defects compared with the 14% that we found in our control embryos (*n*=218; Fig. 3H). These defects were observed most frequently between metaphase and telophase of the cell cycle, and the defects seemed to accumulate during the later syncytial division cycles (Fig. S5). From these experiments, we conclude that *Mms19* has important mitotic functions during the nuclear division cycles 10-13 of the young *Drosophila* embryo.

### Subcellular localization of Mms19::eGFP

**Fig. 4.**
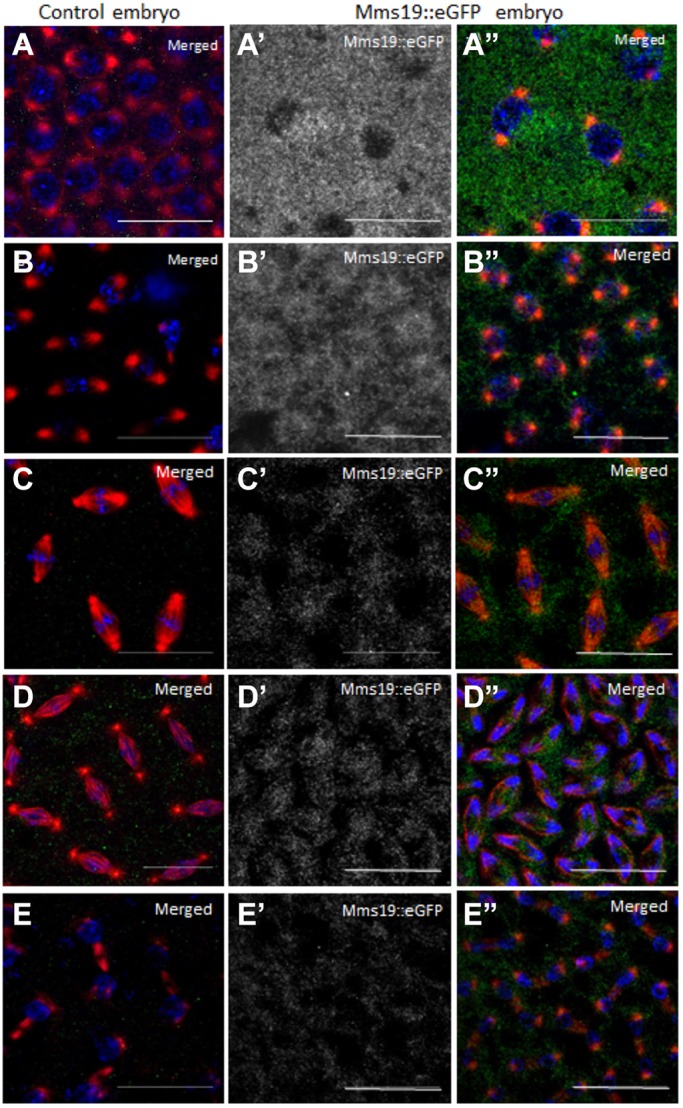
**Mms19::eGFP distribution during the embryonic cell cycle.** Confocal settings were adjusted such that no signal was apparent in the GFP channel when imaging wild-type control embryos lacking GFP (wild-type embryo, A-E). The same microscope settings were then used to image embryos expressing Mms19::eGFP. (A′,A″) Cycle 10 embryo during interphase. (B′,B″) Prophase cycle 11 embryo, showing localization of Mms19::eGFP around the nuclei and also inside them. (C′,C″) Spindle microtubule and nuclear localization of Mms19::eGFP during metaphase 11. (D′,D″) Anaphase 12 embryo. (E′,E″) Cycle 12 embryo in telophase showing the presence of Mms19::eGFP in nuclei and in mid bodies. In merge panels, DNA is in blue, α-Tubulin in red, Mms19::eGFP in green. Scale bars: 20 µm.

We studied the localization pattern of Mms19::eGFP in young embryos, a stage when gene products provided by the mother are active. To ensure normal expression levels, a genomic copy of *Mms19*, tagged with *eGFP*, was expressed maternally from its own promoter and the endogenous *Mms19* gene was inactivated (*Mms19^P/P^*). The first 13 divisions in the *Drosophila* embryo are rapid syncytial divisions that lack G phases and consist of alternating S and M phases. Focusing again on the nuclear division cycles 10-13, a clear Mms19::eGFP expression pattern became apparent (Fig. 4A-E). Interphase Mms19::eGFP signal was strongest and detected primarily in the cytoplasm whereas only a weak signal was seen in the nuclei (Fig. 4A′,A″). Upon entry into mitosis, the GFP signal concentrated around the nuclei with higher signal intensity at the spindle poles. The initially low levels of nuclear GFP signal became progressively higher during prophase (Fig. 4B′,B″). As the nuclei entered metaphase, the signal was present inside the nuclei where it seemed to associate with the spindle microtubules. Additionally, small pockets free of Mms19::eGFP signal had started to form in the cytoplasm between neighbouring nuclei (Fig. 4C′,C″). Anaphase Mms19::eGFP signal was still predominantly in the spindle region, but it seemed to colocalize less with tubulin (Fig. 4D′,D″), a process that continued into telophase (Fig. 4E′,E″). Live imaging of embryos expressing Mms19::eGFP and Jupiter::mCherry (for marking the spindle microtubules) gave similar results (Fig. S6) although the Mms19::eGFP signal observed in live imaging was weaker and bleached more readily. In the light of the various mitotic phenotypes observed in *Mms19* mutant embryos, the dynamic localization pattern of Mms19::eGFP might point towards diverse functions of *Mms19* or towards a function that involves dynamic localization changes.

### Mms19::eGFP interacts with Galla-2 and Xpd in *Drosophila* embryos

Ito et al. (2010) had previously reported the existence of an MMXD complex in human cells and they suggested that this complex might play a role in chromosome segregation. However, the pathways through which this complex functions still remained unknown. To find out more about the molecular mechanisms of the mitotic activity of *Drosophila Mms19*, we first tested whether fly Mms19 interacts with Xpd and Galla-2, the fly homologue of MIP18 (Yeom et al., 2014). For this, we expressed Mms19::eGFP and performed immunoprecipitations (IPs) with extracts from 0- to 2 h-old *Drosophila* embryos. The results shown in Fig. S7A demonstrated that pulling down Mms19::eGFP with an anti-GFP antibody led to the specific co-IP of Xpd and Galla-2 (but not Cdk7), indicating that these complexes are evolutionarily conserved. IPs with extracts from HEK293T cells transfected with constructs of the three *Drosophila* genes *FLAG::Mms19*, *HA::Galla-2* and *xpd* confirmed the interaction between these *Drosophila* proteins (Fig. S7B).

We also used these extracts to perform IPs with an antibody against another polypeptide that forms complexes with Xpd, Cdk7. When Cdk7 IPs were analysed for the presence of Xpd, a much stronger Xpd signal was detected. Although IPs performed with different antibodies cannot be compared quantitatively, the strong difference suggests that Xpd complexes with Cdk7 are more abundant or stable during the first 2 h of embryogenesis. It is also possible that these complexes are present during a longer part of the cell cycle than are complexes containing Xpd and Mms19 (Fig. S7A). Importantly, these co-IPs also showed no evidence that Mms19::eGFP and Cdk7 are present in the same complex, indicating that Xpd interacts only with one of them at the time. Because Xpd forms alternative complexes either with Mms19::eGFP or with Cdk7, it appears that the binding of Xpd to Mms19 and to the Cdk7-CAK complex might be mutually exclusive and that Mms19 and the Cdk7-CAK complex might compete with one another for binding to Xpd. We will discuss the structural basis for such a competition and its implication for the regulation of the CAK activity in the Discussion.

### *xpd* affects expression and localization of Mms19::eGFP

**Fig. 5.**
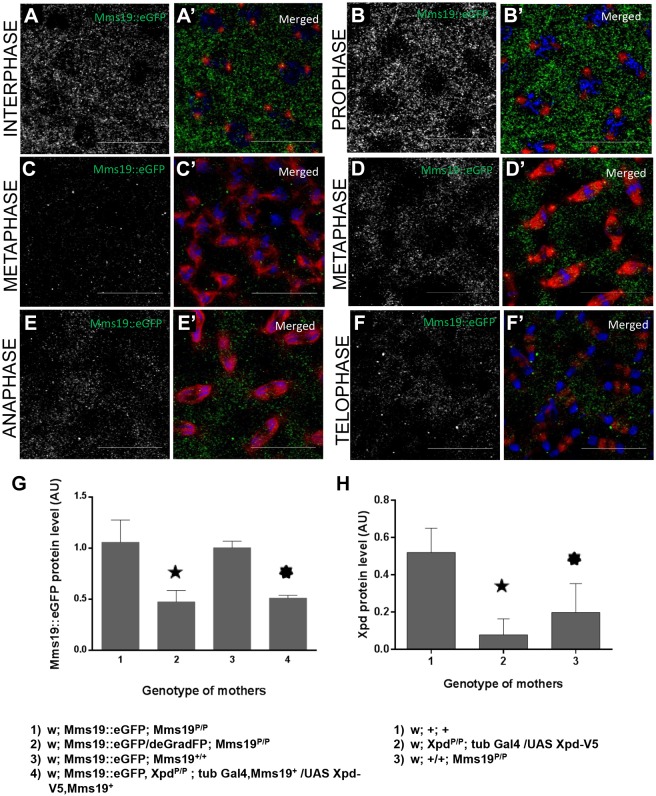
**Mislocalization of Mms19::eGFP in *xpd^eE^* embryos.** Compared with the expression of Mms19::eGFP in wild-type embryos (Fig. 4), the expression in *xpd^eE^* embryos is reduced as is evident from the weaker GFP signal in all phases of the cell cycle. Antibody staining was performed in parallel and settings for imaging were identical. (A,A′) During interphase, the weaker Mms19::eGFP signal was normally distributed. (B,B′) During prophase, the stronger Mms19::eGFP signal around the nuclei was missing/absent, but the protein was still present primarily in the cytoplasm. (C,C′) Embryo with severe defects showing very little Mms19::eGFP expression and no specific localization. (D,D′) In metaphase embryos with mild defects, the Mms19::eGFP expression was not very different from that of the wild type and it localized also to the spindle microtubules. (E-F′) Anaphase (E,E′) and telophase (F,F′) embryos showed reduced Mms19::eGFP expression with loss of specific localization at the spindle microtubules and around the nuclei, respectively. In merge panels, DNA is shown in blue, α-Tubulin in red. (G) Expression of Mms19::eGFP protein was normalized to α-Tubulin. Mms19::eGFP protein levels are reduced to almost half upon knockdown with the deGradFP technique (2, star) compared with controls without knockdown (1, 3) expression. Similarly, reduced Mms19::eGFP levels were also observed in *xpd^eE^* embryos (4, sun). (H) Quantification of Xpd protein levels relative to α-Tubulin in the same sample of embryos derived from mothers with the indicated genotype. Xpd levels are strongly reduced in *xpd^eE^* embryos (2, star) and normalized Xpd levels were also reduced in embryos derived from *Mms19^P/P^* mutant germline clones (3, sun). In G,H, results were from three independent experiments and error bars represent s.d. Scale bars: 20 µm.

To test whether *xpd* is involved in the expression and dynamic localization of Mms19, we studied the localization of Mms19::eGFP in 0- to 2-h-old embryos that express little or no Xpd maternally (*xpd^eE^*; Li et al., 2010) (Fig. 5). Indeed, in the absence of Xpd Mms19::eGFP levels were reduced to almost half (Fig. 5G, bar 4). Consistent with the western blot result we also observed that *xpd^eE^* embryos expressing Mms19::eGFP showed reduced GFP staining intensity under the microscope during all stages of the cell cycle (compare Fig. 5 with the *xpd^+^* wild type in Fig. 4). Because image acquisition and processing were carried out the same way for the panels of these two figures, they can be compared directly. Interestingly, loss of Mms19::eGFP signal in metaphase correlated with the severity of the *xpd^eE^* phenotypes observed in individual *xpd^eE^* embryos (compare strong phenotype in Fig. 5C with mild defects in 5D). Although we cannot rule out the possibility that slight differences in age distribution between the samples with and without Xpd slightly affect the measurement of Mms19 levels (even though embryos were collected in parallel and grown under the same conditions) we conclude that *xpd* is needed for normal Mms19::eGFP levels.

Aside from this quantitative difference, we observed that specific aspects of the dynamic localization pattern were also affected by the lack of Xpd. Although the interphase distribution of Mms19::eGFP was not affected by the lack of Xpd (compare Fig. 5A with Fig. 4A′), the specific localization of Mms19::eGFP around the nucleus seen during wild-type prophase (Fig. 4B′) was not observed (Fig. 5B). Whereas Mms19::eGFP usually concentrates around the spindle during metaphase, it remained more uniformly cytoplasmic in embryos lacking Xpd (compare Fig. 5D with Fig. 4C′). Similarly, the staining adjacent to centrosomes observed in wild-type telophases was lost in *xpd^eE^* embryos and, instead, the cytoplasmic signal seemed more uniform (Fig. 5F). We conclude that *xpd* plays a role in localizing Mms19::eGFP around the nuclear envelope in prophase, in the spindle region in metaphase and around the centrosomes in telophase.

To find out whether Xpd levels also depend on *Mms19* activity, we used *Mms19^P/P^* germline mutant embryos to measure Xpd levels relative to α-Tubulin levels in the same sample. Indeed, western blotting revealed a significant reduction of Xpd levels in *Mms19^P/P^* germline mutant embryos (Fig. 5H). Therefore, Mms19 and Xpd mutually depend on each other for their normal expression and stability.

### Overexpression of CAK restores the diploid larval tissue in *Mms19^P/P^* mutants

Lack of diploid tissues similar to our observations in *Mms19^P/P^* larvae has also been described for *Cdk7* and *Cdk1* loss-of-function mutants (Larochelle et al., 1998; Stern et al., 1993). This similarity suggests that *Mms19* might be acting through the Cdk7-Cdk1 pathway to perform its role in mitosis, even though it did not seem to physically interact with Cdk7 (Fig. S6A). If true, it might be possible to rescue the *Mms19* mutant phenotype partially by overexpressing the CAK complex. To test this pathway, we overexpressed Cdk7, Cyclin H and Mat1 under the control of the *daughterless (da)-Gal4* driver in *Mms19^P/P^* larvae. Amazingly, overexpression of CAK was able to bring back all imaginal discs (Fig. 1C-C″). Even though at least some discs appeared less well-structured than their wild-type counterparts, they reached the normal size. In contrast, *Mms19^P/P^* larvae did not show any disc formation even 14 days after egg laying when they had reached the size of wild-type third instar larvae (Fig. 1A-A″).

**Fig. 6.**
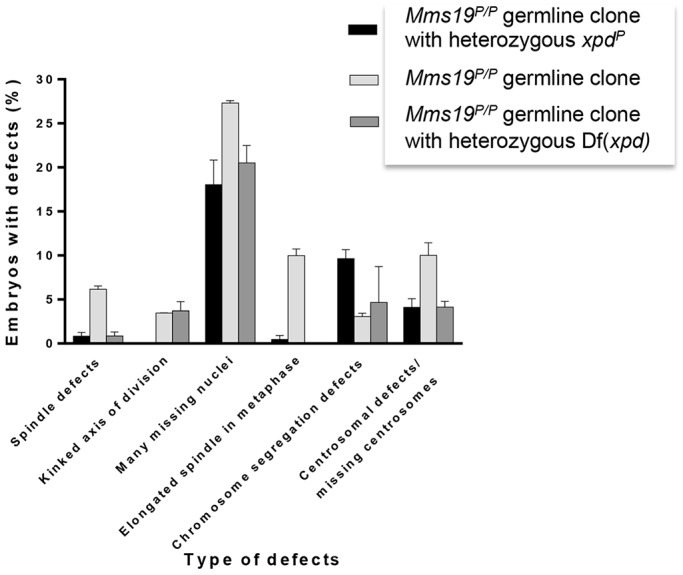
**Reducing the *xpd* dose can rescue the *Mms19* phenotype.** Of the different mitotic phenotypes observed in embryos that do not obtain functional Mms19 from their mothers, all but one can be rescued at least partially by reducing the *xpd^+^* dose using heterozygous *xpd^−/+^* loss-of-function chromosomes. Two independent *xpd^−^* chromosomes were used: *xpd^P^* and Df(*xpd*), a chromosome that has the *xpd* gene deleted. The exceptions were the chromosome segregation defects, which increased under these conditions. Overall, embryos derived from *Mms19^P/P^* germline clones containing two copies of *xpd^+^* showed mitotic phenotypes in 60% (*n*=260) of cycle 10-13 embryos, whereas embryos derived from *Mms19^P/P^* germline clones with only one copy of *xpd^+^* showed such phenotypes only in 30% (*n*=260) of cycle 10-13 embryos with the *xpd^P^* chromosome and 26.8% (*n*=317) with the Df(*xpd*) chromosome. Results were obtained from three independent experiments. Error bars show represent s.d.

Xpd has been shown to repress the CAK activity of Cdk7 (Chen et al., 2003). To understand better the role of the interactions between Mms19 and Xpd, we considered the possibility that Mms19 not only activates or stabilizes Xpd by delivering the Fe-S cluster (Gari et al., 2012; Stehling et al., 2012), but that Mms19 might also prevent Xpd from inhibiting the CAK activity during normal mitosis. If this is true, it might be possible to also rescue the *Mms19* mutant phenotype partially by downregulating *xpd* in the *Mms19^P/P^* background. We tested this hypothesis with two different approaches. First, we reduced *xpd* activity preferentially in the diploid tissue by driving *xpd* RNAi with the *da-*Gal4 driver. This treatment did not increase larval viability, possibly because the knockdown was so severe that lack of Xpd killed the larvae. Replacing the wild-type *xpd^+^* chromosome with either of two unrelated chromosomes carrying an *xpd^−^* loss-of-function mutation (*xpd^P^* and a deficiency for *xpd*) we obtained partial rescue of most of the embryonic phenotypes of *Mms19^P/P^* germline clones, strongly suggesting that reducing the *xpd^+^* dose causes suppression of the *Mms19* phenotypes. Overall, suppression caused the frequency of mitotic phenotypes in embryos in division cycles 10-13 to drop from 60% to 30% (Fig. 6). Interestingly, although most of the different phenotypes were rescued, the frequency of the phenotype ‘chromosome segregation defects’ actually increased in the presence of only one functional *xpd^+^* copy (Fig. 6).

## DISCUSSION

Human and yeast MMS19 are part of the late-acting CIA machinery that facilitates the transfer of Fe-S clusters to their target proteins (Gari et al., 2012; Stehling et al., 2012). Human MMS19 performs this activity as part of a cytoplasmic complex with the other components of the CIA machinery, CIAO1, MIP18 and IPO1. The targets of this machinery are Fe-S cluster-binding proteins that interact with DNA and have functions in DNA repair. One additional *MMS19* study described a potentially unrelated role for *MMS19* and *MIP18* in mitosis because siRNA-mediated knockdown of *MMS19* or *MIP18* led to mitotic defects (Ito et al., 2010). Similarly, reducing the activity of the fly genes encoding the two Mip18 homologues Galla-1 and Galla-2 also led to mitotic phenotypes, suggesting that the mitotic function of *Mms19* is performed by the Mms19/Mip18 complex and that this function is conserved from flies to humans (Yeom et al., 2014). These studies combined led to the interesting question of whether the effects on chromosome segregation and spindle dynamics observed upon *MMS19* knockdown in human cells are an indirect result of its role in the CIA or whether they point to a more direct role of *Mms19* in mitosis.

The function of *MMS19* had so far not been studied in multicellular organisms and this study on *Drosophila Mms19* therefore describes for the first time the essential role of *Mms19* as a mitotic gene. It has this role in cells with different types of cell cycles – the rapid cycles in syncytial embryos, which lack G phases, and diploid larval cells, which show a full cell cycle with G phases. At the organismal level, lack of *Mms19* causes lethality at the larval-to-pupal transition and it results in reduced brain size and in the absence of imaginal discs and other diploid cells (Fig. 1A-A″), a phenotype that has long been considered typical for mitotic genes (Gatti and Baker, 1989; Larochelle et al., 1998; Stern et al., 1993). We were also able to demonstrate directly the mitotic function of *Mms19* by knocking down the Mms19 protein supplied to the zygote by the mother. Reducing Mms19 proteins in young embryos led to several severe mitotic defects in syncytial embryos (Fig. 3).

The subcellular localization of Mms19::eGFP is highly dynamic during the syncytial division cycles (Fig. 4). Mms19::eGFP is localized to the cytoplasm during interphase and previous studies showed that this is where it interacts with the CIA machinery and transfers the Fe-S cluster to target proteins, such as XPD (Gari et al., 2012; Vashisht et al., 2015). During M phase, Mms19::eGFP then enters the nuclear area and it can be observed in the vicinity of the spindle microtubules. Given its mitotic functions and the defects in spindle structure and dynamics observed when *Mms19* activity is reduced, the mitotic localization around the spindle region might be the place where it plays a CIA-independent role in mitosis. In this context, it is also interesting to note that independent studies have found Mms19 associated with Tubulin in extracts from young *Drosophila* embryos (Gallaud et al., 2014).

The mitotic function of *Mms19* and *Galla-2/MIP18* combined with the fact that *Drosophila* and human Mms19 form complexes with Xpd and Galla-2/MIP18 is intriguing because Xpd regulates mitosis through Cdk-activating kinase (CAK) (Chen et al., 2003; Li et al., 2010; Ito et al., 2010; Yeom et al., 2014). We therefore considered the possibility that Mms19 could act through the same pathway as Xpd and CAK to allow cell cycle progression through mitosis. The fact that lack of *Cdk7* (Larochelle et al., 1998) or *Cdk1* (Stern et al., 1993) function causes the same larval phenotype as lack of *Mms19* further supports this view. This suggests that the major function of *Mms19* in mitosis could be to allow CAK to phosphorylate and strongly activate the mitotic kinase Cdk1. We were able to confirm this hypothesis by overexpressing the components of the trimeric CAK complex with the *da-Gal4* driver in *Mms19^P/P^* mutant larvae. This led to remarkable restoration of imaginal disc formation (Fig. 1C-C″) and suggests that the excess CAK is capable of activating the mitotic Cdk1 in the absence of Mms19. From this, we conclude that an important function of *Mms19* is to activate the mitotic kinases or kinase pathways and that the *Mms19* mutant phenotypes, the mitotic defects observed in syncytial embryos and in larval cells, are caused by incomplete activation of the mitotic kinase pathway.

What role could the interaction between Mms19 and Xpd play in mitotic progression? Xpd is able to prevent CAK from activating Cdk1 and there is evidence that Xpd can get redistributed or downregulated at the beginning of mitosis (Chen and Suter, 2003; Chen et al., 2003). Mms19 could therefore act as a regulator of Xpd and prevent it from inactivating the Cdk-activating kinase activity of the trimeric Cdk7/CycH/Mat1 (CAK) complex as cells enter mitosis. The proposed pathway predicts that it might be possible to rescue the *Mms19* mutant phenotype not only by overexpression of CAK, but possibly also by reducing the Xpd levels in *Mms19* mutants. Indeed, most of the defects observed in young embryos lacking functional Mms19 could be partially rescued by exchanging one wild-type *xpd^+^* chromosome with either of two unrelated chromosomes that are *xpd^−^*. The fact that two unrelated *xpd^−^* chromosomes cause this suppression strongly suggests that it is indeed the reduction of the *xpd* dose that causes the suppression and not another mutation that happens to be present on both chromosomes. We therefore found good evidence that the suppression is indeed caused by the reduction of functional *xpd*. Based on these results, we propose a pathway for the mitotic function of *Mms19*. As cells enter mitosis, Mms19 prevents Xpd from inhibiting the Cdk-activating kinase activity of the CAK complex. The fully active CAK then performs its function during the cell cycle and phosphorylates Cdk1 in its T-loop, an essential step in the activation of Cdk1.

Although the overexpression of the three CAK components can rescue imaginal disc formation, leading to outgrown discs, the structure of the discs appeared abnormal under the light microscope. Interestingly, suppression was possible with the *da-Gal4* driver, but not with *actin-Gal4* or *6985-Gal4*. This points to the importance of the amount, tissue specificity and timing of CAK expression for the division of these diploid cells. Aside from proper fine-tuning of CAK activity, lack of the cytoplasmic function of *Mms19* in activating and stabilizing Xpd and other Fe-S-containing proteins (Gari et al., 2012; Stehling et al., 2012; Vashisht et al., 2015) might also contribute to the defects still observed in the rescued discs.

A recently published article could provide the structural basis for the dual role of Mms19 towards Xpd that we are proposing. There is good evidence that Fe-S cluster proteins receive the Fe-S cluster by binding to the C terminus of MMS19 (Odermatt and Gari, 2017). Stable interaction with the C terminus also requires the other CIA proteins, MIP18 and CIAO1. Although it is not yet clear whether Xpd binds the C terminus of MMS19, Xpd was shown to bind strongly to the N terminus of MMS19, even in the absence of other CIA proteins. It will be interesting and important to find out whether two different binding sites are used for the two different functions.

Previous studies found that the interaction of XPD with MMS19 and with core TFIIH components are mutually exclusive (Ito et al., 2010; Vashisht et al., 2015). Similarly, we also found that the interactions of *Drosophila* Xpd with the Cdk7-CAK complex and with Mms19::eGFP are mutually exclusive (Fig. S7A). It thus appears that Mms19 binding to Xpd competes with the interaction of Xpd with the core TFIIH component p44 and the CAK complex. Our model therefore proposes that Mms19 binding releases Xpd from core TFIIH by releasing it from p44 and that it additionally releases the trimeric CAK complex from Xpd, allowing it to become an active Cdk-activating kinase. Interestingly, the regions on Xpd needed for the binding of the three different proteins have been mapped and they seem to explain such a competition model (summarized by Stettler et al., 2014). The region in XPD spanning amino acids 277-286 is essential for its interaction with MMS19 (Vashisht et al., 2015) and it is part of the ARCH domain (amino acids 245-443), which is also required for its stable association with the trimeric CAK complex (Sandrock and Egly, 2001). Similarly, the binding sites for Mms19 and p44 are at least adjacent if not overlapping (Sandrock and Egly, 2001; Dubaele et al., 2003; Stettler et al., 2014). Steric interference and direct competition for binding sites might therefore provide the structural basis for the exclusive interaction of XPD with either Mms19 or the CAK (and TFIIH) complex. This mechanism could allow Mms19 to sequester Xpd from the CAK complex during mitosis.

Our model describes how the Mms19-Xpd interaction causes fluctuations of cellular CAK activity. This mechanism might spatially and temporally regulate the rapid nuclear division cycles of the embryo. Edgar and co-workers have carefully analysed the different modes of regulation of Cdk1 activity during the early embryonic cycles of *Drosophila* development and they found that only two Cdk1 isoforms are present during the first 13 cycles. Whereas the inhibitory phosphorylations at the N terminus, known to control Cdk1 activity in later embryonic stages, are not detectable during these early division cycles, the non-phosphorylated isoform and the T-loop phosphorylated isoform, which has become phosphorylated and activated by CAK, are present (Edgar et al., 1994). During the first eight nuclear cycles, fluctuations of the ratio of these two isoforms are not apparent on western blots, but local fluctuations might still happen. However, from cycle 9 onwards such changes could be demonstrated using carefully staged embryos. From this stage onwards, cell cycle phase-dependent differences become progressively more pronounced with the T-loop phosphorylated isoform being more abundant during M phase and reduced during interphase. By cycle 13, the interphase reduction was so strong that this isoform was barely detectable (Edgar et al., 1994). This developmental window, in which the fluctuation of Cdk1 T-loop phosphorylation becomes progressively stronger, coincides with the window in which zygotic transcription increases progressively, too (reviewed by Lee et al., 2014; Tadros and Lipshitz, 2009). So far, the mechanism for this cell cycle phase-dependent change in Cdk1 activation has remained unknown. Our model can explain these observations and it requires neither synthesis nor degradation of regulatory proteins. Once Xpd binds to CAK in interphase, it would recruit CAK into TFIIH for the transcription activity and at the expense of its Cdk1-activating activity (Li et al., 2010; Fisher et al., 2012). Once Mms19 binds to Xpd, the trimeric CAK complex would be released from Xpd and TFIIH, shutting down transcription, but enabling freed CAK to phosphorylate the Cdk1 T-loop and activate the M-phase kinase during the M phase.

## MATERIALS AND METHODS

### DNA constructs and transgenic flies

For the *Mms19::eGFP* construct, the *Mms19* gene region along with 432 bp of 5′ and 372 bp of 3′ flanking sequences were amplified from genomic fly DNA of the OregonR fly strain. The *eGFP* open reading frame (ORF) was amplified from a plasmid (Heim et al., 1995). The sequences were cloned into the pw+SNattB vector (Koch et al., 2009) with the *eGFP* ORF at the C-terminal end of the *Mms19* ORF*.* The constructs for overexpressing the CAK complex were obtained by individually cloning the coding sequences of *Cdk7*, *Mat1* and *Cyclin H* amplified from fly cDNA in the pUAST-attB vector (Bischof et al., 2007). Transgenic stocks were established with the attP landing platform 58A (for *Mms19::eGFP*), 22A3 (for *Cdk7*), 65B2 (for *Mat1*), 86F8 (for *Cyclin H*) and the ΦC31 integration system (Bischof et al., 2007). DNA constructs used for transfection into HEK293T cells were made by cloning the ORF of fly *Mms19*, *Galla-2* and *xpd* from OregonR cDNAs into the pCS2 vector (Addgene).

### Fly stocks

The following fly lines were obtained from the Bloomington Stock Center:y1 w*; P{*neoFRT*}82B *Sb1*/TM6(#2051),w*; P{*neoFRT*}82B P{*ovoD1-18*}3R/ *st1 βTub85DD ss1 es*/TM3, *Sb1* (#2149),*da-GAL4* (#55851), *y1 w67c23*; P{*EPgy2*}*Mms19EY00797*/TM3, *Sb1 Ser1* (#15477; referred to as Mms19P), w;Df(3R)ED5147/TM6C, *Sb* (#8967 *Mms19* deficiency line in which 82E7-83A1 is deleted). *xpdeE* embryos were obtained as described previously (Li et al., 2010).

### Immunostaining and image analysis

Embryo staining was described previously (Chen et al., 2003). Primary antibodies used for staining were: rabbit anti-CNN (1:500; Heuer et al., 1995), rabbit anti-GFP (1:300; 210-PS-1GFP, ImmunoKontact; pre-absorbed before use) and mouse anti-α-Tubulin, clone DM1A (1:500; T9026, Sigma). DNA was visualized with 2.5 µg/ml Hoechst 33258 (Molecular Probes). The secondary antibodies were anti-rabbit and anti-mouse Oregon Green 488 and Alexa Fluor 594 (1:1000; O-11038 and A-11032, Molecular Probes). All imaging of fixed samples was performed using Leica TCS-SP8 confocal microscopes. The images were analysed using Fiji software. For embryos stained with anti-GFP antibody, the background was removed by setting minimal pixel value to 50 and the maximal one to 255. This was done for both control embryos and embryos expressing *Mms19::eGFP*.

### Live imaging

Flies for live imaging were maintained at 25°C and allowed to lay eggs on apple juice plates for 3 h before collection started. Embryos were then dechorionated with 2.5% bleach, washed well with water, glued to the coverslip using heptane glue and then covered with Voltalef oil 3S (VWR). Live imaging was performed with a Leica TCS-SP8 confocal microscope in a chamber maintained at 25°C. The images were then *z*-projected and de-noised using Fiji.

### Culturing and transfection of HEK293T cells

The HEK293T cells were maintained in culture at 37°C in 75 cm^2^ flasks using DMEM medium with 10% foetal calf serum. Transfections were carried out 15 h after seeding (∼70% confluent) using polyethylenimine (PEI). The ratio of PEI to DNA was 1:3. Cells were harvested 48 h after transfection.

### Immunoprecipitation and western blotting

For immunoprecipitation with embryo extract, 1 g of embryos per sample was homogenized in 1 ml lysis buffer [25 mM HEPES pH 7.4, 150 mM NaCl, 0.5 mM EDTA pH8.0, 1× protease inhibitor cocktail (Roche), 1× phosphatase inhibitors, 0.5% NP-40, 2 mM MgCl_2_, 2 mM dithiothreitol]. The lysate was incubated at 4°C for 6 h with proteinG beads (GE Healthcare), pre-coated with monoclonal anti-GFP antibody (gift from Anne Marcil, National Research Council, Montréal, Canada; 1:300). The beads were washed three times in lysis buffer before the protein was eluted in Lämmli buffer. Immunoprecipitation experiments with HEK293T cells were carried out 48 h after transfection. The lysis buffer used for cells contained 20 mM Tris pH 7.4, 150 mM NaCl, 2 mM EDTA pH 8.0, 50 mM NaF, 10% glycerol, 1% Triton X-100, 1× protease inhibitor cocktail (Roche) and 100 mM phenylmethylsulphonyl fluoride. The supernatant was then incubated overnight with proteinG beads (GE Healthcare), pre-coated with rat anti-HA antibody (Roche). Proteins eluted in Lämmli buffer were then run on SDS-PAGE gels for detection. Primary antibodies used for western blot detection were: rabbit anti-GFP (1:1000; 210-PS-1GFP, ImmunoKontact), rat anti-HA (1:1000; 11867423001, Roche), rabbit anti-FLAG (1:1000; SC.807, Santa Cruz), rabbit anti-Xpd (1:500; Chen et al., 2003), monoclonal anti-alpha-Tubulin (1:1000; AA4.3, Developmental Studies Hybridoma Bank), rabbit anti-Galla-2 (1:5000; Yeom et al., 2014), monoclonal anti-Cdk7 (1:20; Larochelle et al., 1998), rabbit anti-pCdk1(Thr161) (1:1000; 9114, Cell Signaling Technologies) and rabbit anti-Cdk1(PSTAIRE) (1:1000; 06-923, Sigma/Merck). Secondary antibodies used were horseradish peroxidase-conjugated antibodies (1:10,000; NA934V, NA931V, NA935V, GE Healthcare). The blots were analysed using Fiji software.

For western blots with pCdk1 antibodies, diploid tissues from 50 third instar larvae were used per sample. The samples were homogenized in Lämmli buffer containing protease inhibitors and phosphatase inhibitors. The SDS-PAGE gels used to resolve the samples were prepared with 30% T:1.67% C acrylamide stock solution using piperazine di acrylamide (BioRad) as the cross linker to resolve better the phosphorylated isoforms of Cdk1 (Larochelle and Fisher, 2005).

## Supplementary Material

Supplementary information
