# Supplementary material for: Mms19 is a mitotic gene that permits Cdk7 to be fully active as a Cdk-activating kinase
Source: Development. 2018 Jan 15;145(2):dev156802. doi: 10.1242/dev.156802 (PMC5825849; doi:10.1242/dev.156802)
Supplement: Supplementary information [file develop-145-156802-s1.pdf]

Supplementary Figure S1

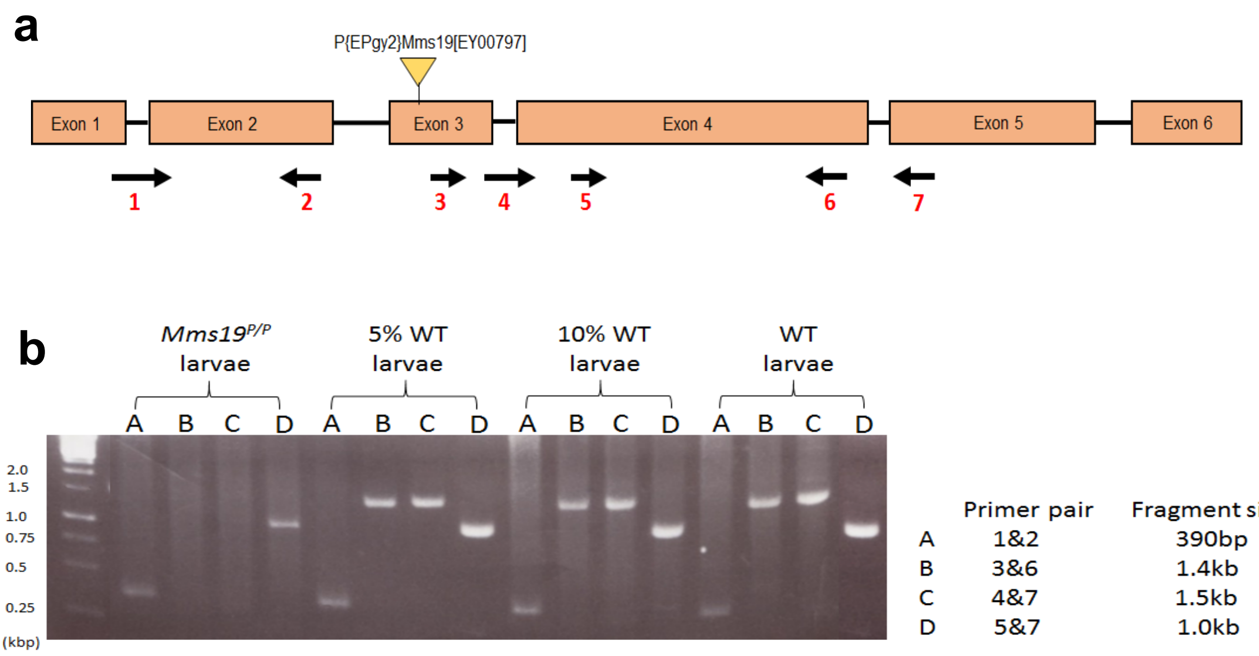

	Primer pair	Fragment size
A	1&2	390bp
B	3&6	1.4kb
C	4&7	1.5kb
D	5&7	1.0kb

RT-PCR results of *Mms19*$^{P/P}$ larvae and WT larvae. (a) A schematic diagram of the *Mms19* gene indicating the position of Exons, P-element insertion and the primer positions used for the RT-PCR. **(b)** Agarose gel image of DNA fragments amplified by primers shown in (a) from cDNA of WT and *Mms19*$^{P/P}$ larvae. *Mms19*$^{P/P}$ larvae contain less than 2-5% transcripts of the *Mms19* exons 4 and 5 (fragment D) and no transcripts containing Exon 3 could be detected. The *Mms19*$^{P/P}$ 5' end fragment A (390bp, exon 2) appears also slightly weaker than the different WT ones.

.

Development • Supplementary information

Supplementary Figure S2

a *w ; Mms19::eGFP/SM1; $Mms19^{P}$*/TM3 Sb ✕ *w ; Mms19::eGFP/SM1 ; $Mms19^{P}$* / TM3 Sb

↓ F1 offspring

Mms19::eGFP copies[2]	1	2	1 or 2	Total frequency
**Genotype[1]**	$Mms19^P/Mms19^P$	$Mms19^P/Mms19^P$	$Mms19^P$/TM3 Sb	
**Expected frequency**	22.2%	11.1%	66.6%	99.9%
**Observed frequency**	14.6%	8.7%	76%	99.3%

b

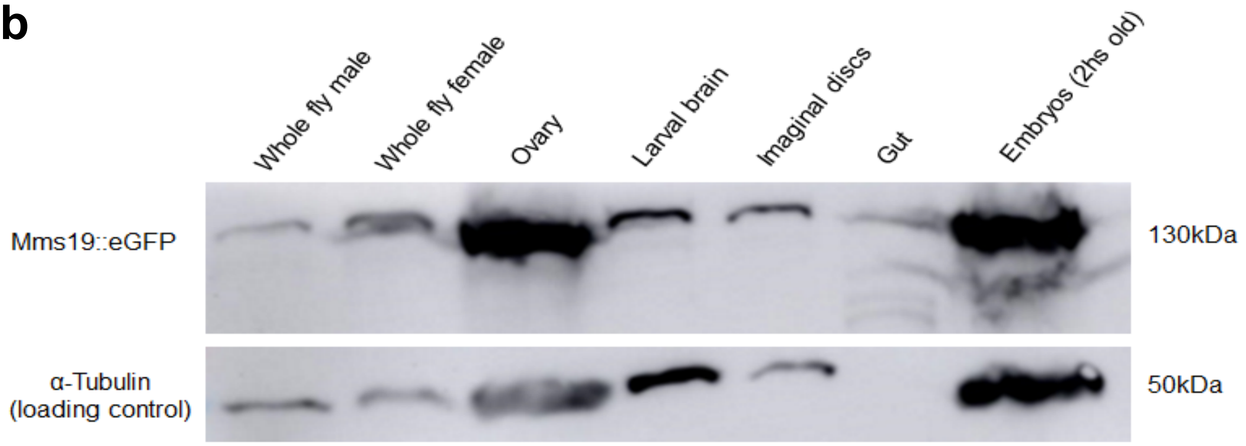

(a) ***Mms19::eGFP rescues $Mms19^{P/P}$.*** **(a)** The rescue cross is shown on top and the frequency of expected and actually identified eclosed flies of the different genotypes is shown in the table below. ([1]) Shows the offspring genotype of the 3rd chromosome. ([2]) shows the number of *Mms19::eGFP* alleles on the 2nd chromosome of the offspring (2: homozygous, 1: heterozygous transgene over SM1 balancer). Note that SM1/SM1 and TM3, Sb/TM3, Sb are lethal. (N=1402). (**b**) Expression of the Mms19::eGFP fusion protein in different fly tissues. Anti-GFP Western blot showing Mms19::eGFP protein expression in the different stages and tissues indicated. Mms19::eGFP runs at 130kDa and the loading control α-Tubulin at 50kDa. Gut samples may be degraded.

Supplementary Figure S3

maternal germ line clone		paternal genotype	larval hatching rate
y w hsFLP/y w; neoFRT^{82B}*, Mms19*^{P}*/neoFRT*^{82B}*, Mms19*^{P}	×	wild type males	**24.5%**
y w hsFLP/y w; neoFRT^{82B}*, Mms19*^{P}*/neoFRT*^{82B}*, Mms19*^{P}	×	*hsFLP/Y ; neoFRT*^{82B}*, Mms19*^{P}*/FRT*^{82B}*, Ovo*^{D}	**10.5%**

Partial paternal rescue of the maternal loss-of-*Mms19 phenotype*. Larval hatching rate of *Mms19*$^{P/P}$ germline mutants. Virgins producing *Mms19*$^{P/P}$ germline clones crossed to wild-type males resulted in offspring that showed a larval hatching rate of 24.5%. This rate was reduced to almost half (10.5%) when their fathers had only one wild-type allele of *Mms19 (Mms19*$^{P/+}$*).*

Supplementary Figure S4

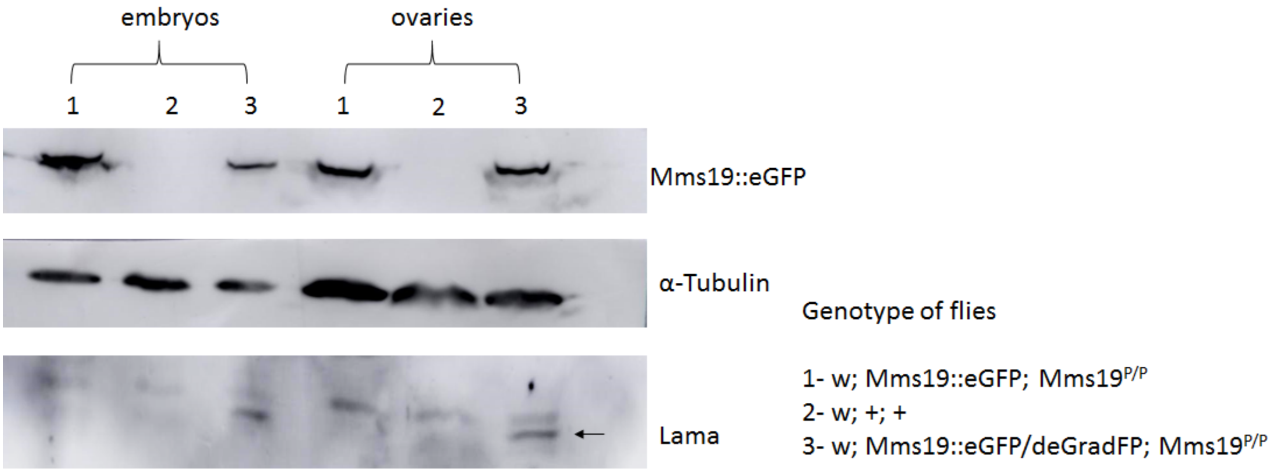

Mms19::GFP levels are reduced in embryos expressing the deGradFP. In ovaries the fusion protein remains more stable. Western blots of extracts from embryos and ovaries of the maternal genotypes indicated. αTubulin served as loading control. Even though some Lama deGradFP is expressed during oogenesis, Mms19::eGFP seems to remain stable. Note that the loading control shows that there is more material loaded on the ovary lanes.

Supplementary Figure S5

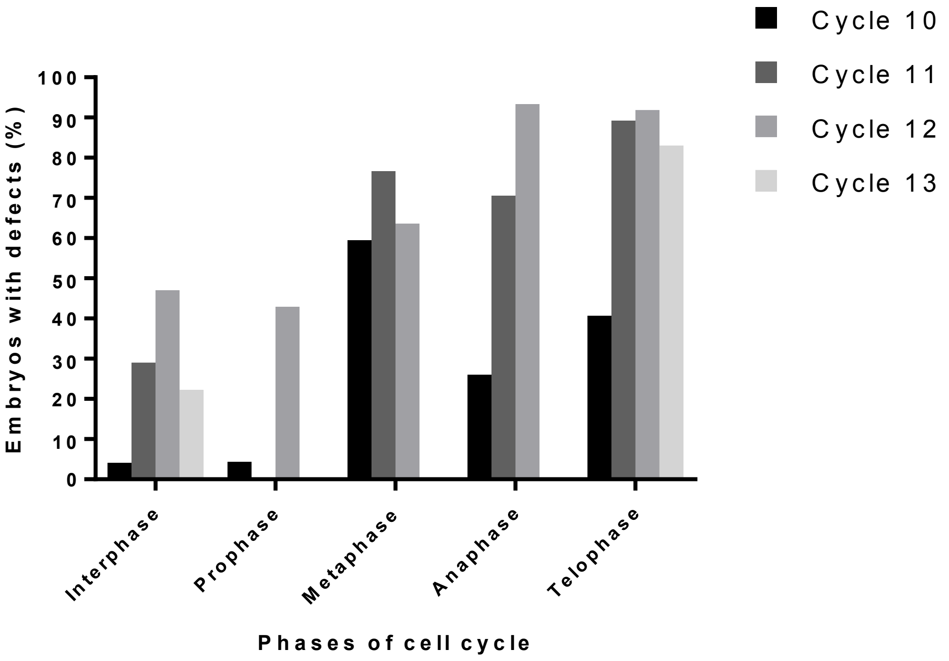

Appearance of defects caused by knockdown of the Mms19::eGFP protein in young embryos. Comparison of the first appearance of all mutant defects observed during the different cell cycle phases of nuclear cycles 10-13. The only defect observed in interphase was the ‘large areas without nuclei’. About 60-70% of embryos in metaphase, anaphase and telophase showed one or more defect with cycle 11 and 12 being the most affected cycles.

Supplementary Figure S6

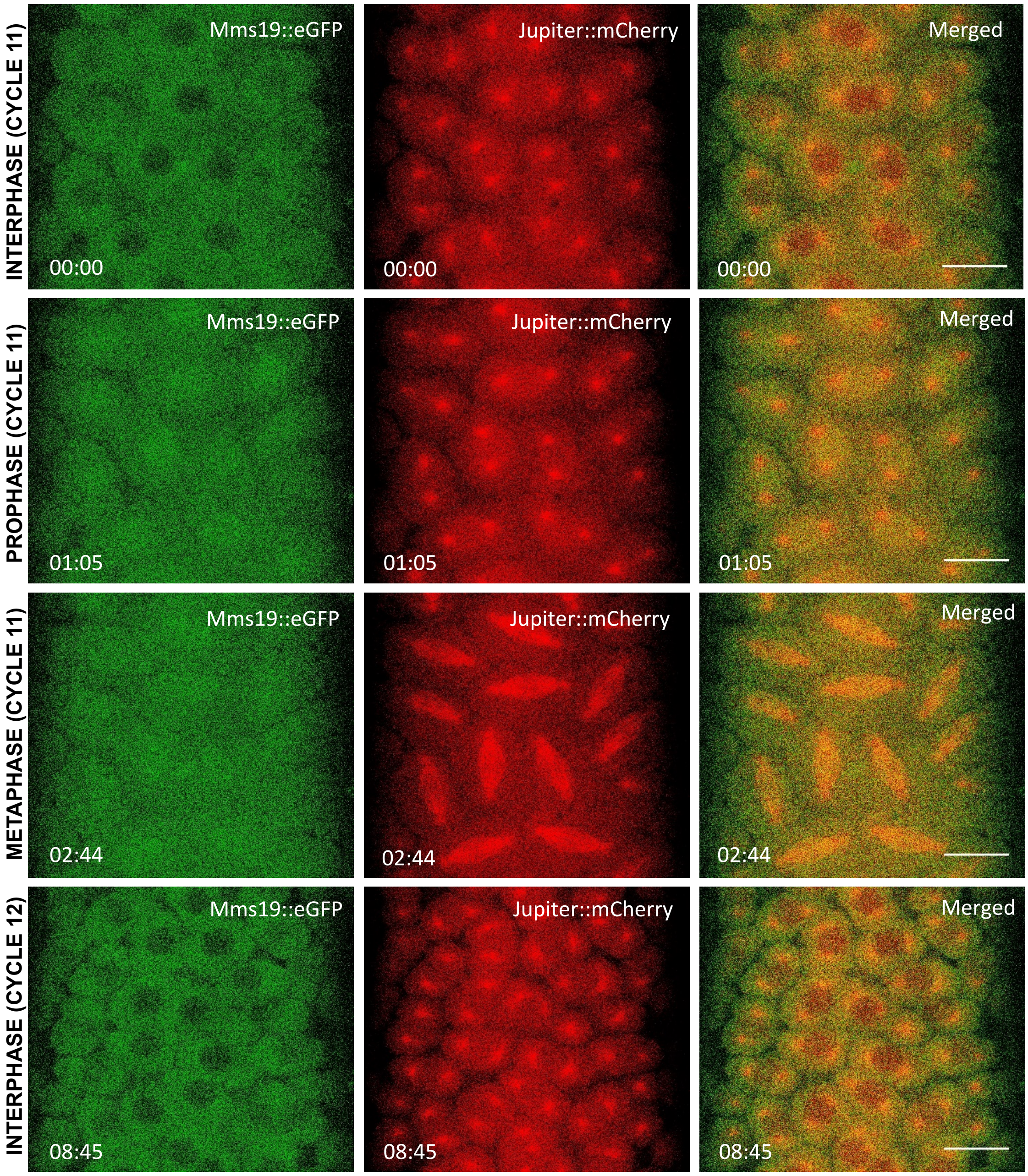

Live imaging by confocal microscopy of embryo expressing Mms19::eGFP and Jupiter::mCherry. Stills from the live movie showing the signals throughout nuclear cycle 11 and into nuclear cycle 12. Time is indicated in min:sec and the scale bar represents 15µm.

Supplementary Figure S7

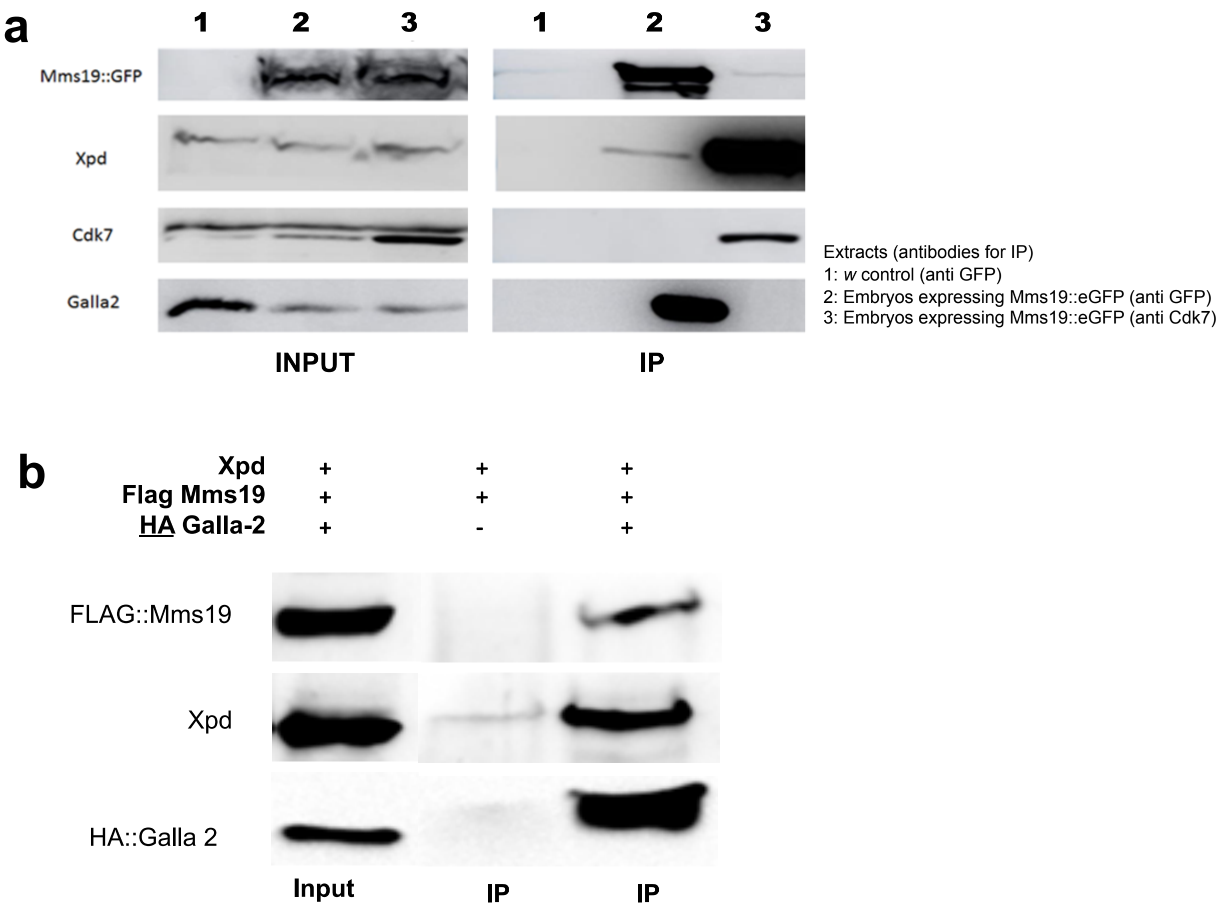

Mms19::eGFP protein complexes with Galla-2 and Xpd. (a) Xpd and Galla-2 are co-immunoprecipitated with an anti GFP antibody (2) when using extracts from young *Drosophila* embryos which express a transgenic Mms19::eGFP under the control of the endogenous *Mms19* promoter (2, 3). **(b)** co-IP with anti-HA antibodies of extracts from HEK293T cells transfected with FLAG::Mms19, HA::Galla-2 and Xpd also showed a pull down of FLAG::Mms19 and Xpd with HA::Galla-2. The Drosophila genes were used for these constructs. + and - above the Figure indicate whether these constructs were transfected.
